# Automation of cleaning and reconstructing residential address histories to assign environmental exposures in longitudinal studies

**DOI:** 10.1093/ije/dyz180

**Published:** 2020-04-15

**Authors:** Daniela Fecht, Kevin Garwood, Oliver Butters, John Henderson, Paul Elliott, Anna L Hansell, John Gulliver

**Affiliations:** 1 UK Small Area Health Statistics Unit, MRC-PHE Centre for Environment & Health, Imperial College London, London, UK; 2 Avon Longitudinal Study of Parents and Children, University of Bristol, Bristol, UK; 3 Institute of Health and Society, Newcastle University, Newcastle upon Tyne, UK; 4 Imperial College Healthcare NHS Trust, London, UK; 5 Centre for Environmental Health and Sustainability, George Davies Centre, University of Leicester, Leicester, UK

**Keywords:** Residential mobility, exposure measurement error, air pollution, cohort studies, reproductive health, pregnancy

## Abstract

**Background:**

We have developed an open-source ALgorithm for Generating Address Exposures (ALGAE) that cleans residential address records to construct address histories and assign spatially-determined exposures to cohort participants. The first application of this algorithm was to construct prenatal and early life air pollution exposure for individuals of the Avon Longitudinal Study of Parents and Children (ALSPAC) in the South West of England, using previously estimated particulate matter ≤10  µm (PM_10_) concentrations.

**Methods:**

ALSPAC recruited 14 541 pregnant women between 1991 and 1992. We assigned trimester-specific estimated PM_10_ exposures for 12 752 pregnancies, and first year of life exposures for 12 525 births, based on maternal residence and residential mobility.

**Results:**

Average PM_10_ exposure was 32.6  µg/m^3^ [standard deviation (S.D.) 3.0  µg/m^3^] during pregnancy and 31.4 µg/m^3^ (S.D. 2.6  µg/m^3^) during the first year of life; 6.7% of women changed address during pregnancy, and 18.0% moved during first year of life of their infant. Exposure differences ranged from -5.3  µg/m^3^ to 12.4  µg/m^3^ (up to 26% difference) during pregnancy and -7.22  µg/m^3^ to 7.64  µg/m^3^ (up to 27% difference) in the first year of life, when comparing estimated exposure using the address at birth and that assessed using the complete cleaned address history. For the majority of individuals exposure changed by <5%, but some relatively large changes were seen both in pregnancy and in infancy.

**Conclusions:**

ALGAE provides a generic and adaptable, open-source solution to clean addresses stored in a cohort contact database and assign life stage-specific exposure estimates with the potential to reduce exposure misclassification.

**Key Messages**

- Longitudinal birth cohort studies often assign environmental exposure during pregnancy based on residential address of the mother at the time of birth which, depending on the pollutant under study, might introduce exposure misclassification.
- We developed an ALgorithm for Generating Address Exposures (ALGAE), a generic, automated process for assigning life stage-specific environmental exposures to birth cohort participants using the cohort contact database.
- We applied ALGAE to assign previously modelled spatiotemporal high-resolution air pollution exposure to ∼14 000 pregnant women recruited as part of the Avon Longitudinal Study of Parents and Children (ALSPAC) birth cohort.
- The successful implementation of ALGAE to ALSPAC demonstrates its potential to reduce exposure misclassification in birth cohort studies.
- Its generic code base makes ALGAE re-usable for other cohort studies, providing an accessible and low-cost means to enhance cohort studies with environmental exposure data.

## Introduction

Longitudinal birth cohort studies provide an important resource to study the onset and development of disease associated with pre- or postnatal environmental exposures through childhood into adulthood.^1–3^ Certain phases of rapid human development, including pregnancy and the first year of life, have been studied in relation to exposure to environmental pollutants and adverse health outcomes.^4–9^ The first and last trimesters of pregnancy, for example, have been identified as key air pollution exposure stages associated with preterm birth and smallness for gestational age, respectively.^10^^,^^11^ Most of these studies assign environmental exposure during pregnancy based on a single point in space and time, such as the residential address of the mother at the time of birth. Residential mobility during pregnancy, however, is common, varying between 10% and 30% according to a recent review.^12^ There is potential for large exposure misclassification from using address at birth or having inaccurate or incomplete address histories, depending on both the characteristics of the move (for example, from city to rural village or within a city)^13^ and the spatial and temporal variability of the pollutant under study.^14^ Ignoring residential changes during pregnancy could, therefore, result in under- or overestimation of effect sizes in epidemiological studies using birth cohorts.

Some countries collect detailed information on residential mobility as part of national registries. In a study on maternal exposure to air pollution and birthweight in Oslo, for example, routinely collected information on residential address was linked to records from the Medical Birth Registry of Norway to account for residential mobility.^15^ Most countries, however, do not maintain such detailed address records of their residents. Cohort studies do not routinely collect residential address histories of their participants either and might need to collect such information retrospectively, for example via person- or computer-assisted phone interviews.^13^^,^^14^^,^^16^^,^^17^ Such retrospective data collections are resource intensive and may be prone to recall bias.^12^

An often overlooked alternative is the use of cohort contact databases—administrative systems set up to audit current addresses of cohort participants. Administrative systems have the advantage that address information is readily available in an electronic format, which allows the gathering of mobility data for large cohort populations without the resources needed for individual data collection. Deriving residential address histories from administrative systems, however, can be challenging. Contact databases are typically designed to audit current addresses, not to track past ones. This means that addresses are usually updated in the database whenever cohort members notify the cohort study of any address changes. Such data management systems are set up to create a new record in the database with a time stamp for the date at which the address was changed in the database, importantly not the date the cohort member moved. Instead of updating existing records, new records are commonly created every time changes are made to the address database, including the correction of errors such as spelling mistakes or adding additional information to an address. In order to reconstruct address histories from contact databases, address records need to be cleaned in a systematic way such that cohort members are recorded as resident at only one location on any given day.

Here we explore the use of a contact database to reconstruct residential history for assigning environmental exposures. We developed an ALgorithm for Generating Address Exposures (ALGAE), a generic, automated process for assigning life stage-specific environmental exposures to cohort participants. We demonstrate this application for an English birth cohort study, the Avon Longitudinal Study of Parents and Children (ALSPAC). We constructed pregnancy trimester- and first year of life-specific exposure estimates based on previously modelled spatially and temporally detailed particulate matter of diameter ≤10  µm (PM_10_) concentrations^18^ at: (i) residential address at birth; and (ii) using reconstructed address histories for each participant to account for mobility during pregnancy and the infants’ first year of life; and we then compared the differences in PM_10_ concentrations between these two methods.

## Methods

ALSPAC, a prospective observational study, is one of the best-characterized birth cohort studies in the world.^19^ It was set up to explore modifiable influences on health across the life course. Centred on the city of Bristol in the South West of England, ALSPAC recruited 14 541 pregnant women with expected dates of delivery between 1 April 1991 and 31 December 1992. This resulted in 14 062 live-born children, of whom 13 985 survived to the end of the first year of life.^19^ Those children have been followed up multiple times and follow-up is still ongoing.

Using a bespoke geocoding algorithm and Ordnance Survey’s AddressBase Plus^©^, we geocoded all residential addresses held in the ALSPAC contact database (*n *=* *45 771), allowing us to assign geographical coordinates to 96.2% of addresses (*n *=* *40 446). We restricted our study to children who did not move outside the original ALSPAC study area which was the extent of the air pollution modelling domain (1333 km^2^), resulting in 36 986 addresses for which we modelled daily air pollution concentrations.

The air pollution modelling is described in detail elsewhere.^18^ In brief, daily average PM_10_ concentrations were modelled for all maternal residential addresses of ALSPAC mothers and their children, between the estimated date of conception of the first baby born in 1991 (1 August 1990) until the end of the first year of life of the last baby born in 1992 (31 December 1993). Dispersion models were used to separately model local (i.e. traffic, housing, industry) and regional (i.e. long-range transport) anthropogenic particulate sources, and added a time-invariant constant to reflect background, non-anthropogenic sources. We focused on PM_10_, although particulate matter of diameter ≤2.5  µm (PM_2.5_) might be a more relevant pollutant in terms of birth outcomes^11^; but such measurements were not available before 2008, which was outside our study period.

In order to assign daily exposure values to ALSPAC participants, we developed ALGAE to systematically clean the addresses stored in the ALSPAC contact database and account for temporal gaps or overlaps between successive address periods that might be present. Having assigned daily exposure estimates to each participant, based on the clean address history, ALGAE then aggregated daily exposures for each pregnancy trimester (T), early infancy (EI, 0–6 months) and late infancy (LI, 7–12 months) accounting for residential mobility.

ALGAE is an automated PostgreSQL script (doi: 10.5281/zenodo.1303960) that runs with PostgreSQL v9.3. ALGAE is freely available under the open source license GPL v3.0 on GitHub [https://smallareahealthstatisticsunit.github.io/algae/] and is extensively documented.^20^ Here we cover some of the key aspects of the process.

The temporal boundaries of life stages were based on date of birth (DoB) and date of conception (DoC), where DoC is defined as DoB−(7 x gestation age at birth in weeks)−1 day, as shown in Table 1. For some premature births, the third trimester (T3) was non-existing and the second trimester (T2) overlapped with days in EI. We fixed these overlaps by deleting T3 and computed the end date of T2 as: DoB−1 day. The ALGAE code clearly highlights the trimester calculations, so modifications to the temporal boundaries of life stages can easily be made.

**Table 1.** Start and end dates of life stages as calculated by ALGAE

Life stage	Description	
	Start date	End date
Pregnancy (P)	DoC^a^	DoB
Trimester 1 (T1)	DoC	DoC + 92 days
Trimester 2 (T2)	DoC +93 days	DoC + 183 days
Trimester 3 (T3)^a^	DoC + 184 days	DoB - 1 day
Early infancy (EI)	DoB	DoB + 6 months – 1 day
Late infancy (LI)	DoB + 6 months	DoB + 12 months – 1 day

a Date of Conception (DoC): Date of Birth (DoB)−(7 x gestation age at birth in weeks)−1 day.

**Figure 1.**
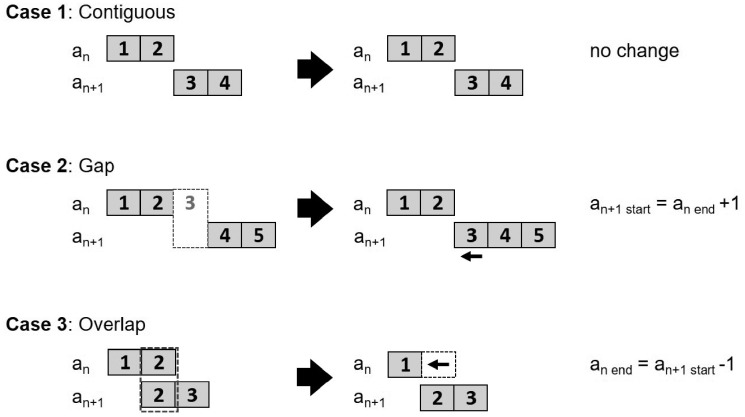
ALGAE’s conditions for cleaning address periods (*a_n_*) derived from a contact database showing cases of: (i) contiguous address periods with complete information of address start (*a_n start_*) and end (*a_n end_*) dates; (ii) gaps in address periods where 1 or more days are missing; and (iii) overlap in address periods where one or more addresses are recorded for the same day for the same individual. In case of gaps or overlaps in address periods, ALGAE favours preserving the start date of address periods over end date.

The ALSPAC contact address database records each instance of change of address, not when study members began living at an address. The ALGAE protocol, therefore, favours preserving start dates over end dates when gaps and overlaps are encountered, assuming the start date of an address period to be a stronger and more reliable signal of location than the end date. This assumption is based on the fact that in an administrative system, start dates will likely correspond to time stamps but end dates will likely be imputed in relation to start dates. In case of gaps and overlaps in address periods, ALGAE therefore imputes start dates with DoC and missing end dates with the current date. Figure 1 illustrates the three scenarios: temporally contiguous address periods (Contiguous); gap between two address periods (Gap); overlap of two address periods (Overlap).

In addition, we corrected address periods if we could not assign geographical coordinates to an address because the address was unknown or fell outside the study area. Corrections were done only if the address period was immediately followed by an address period with a valid geocode, and the duration of the address period with invalid geocode did not overlap by more than 25% of days with any live stage. Such address periods were corrected by allowing the address period after it to subsume it. This process places each individual for each day of each life stage at one address. ALGAE could then assign life-stage specific exposures based on the modelled daily PM_10_ concentrations for each location and computed mean, median and cumulative exposures across each life stage using the address history.

We compared estimated exposures obtained using the reconstructed address histories with those obtained using residence at birth for the whole duration of pregnancy and infancy (i.e. as often used in epidemiological studies), to explore the impact of reconstructing address histories on potential exposure misclassification. We used descriptive statistics, R^2^ and Spearman’s correlation to describe the relationship between the two different exposure estimates.

## Results

Of all address periods processed, ALGAE corrected the start and end dates of 69% of records; 19% of address periods had more than one date changed. Based on the corrected address periods, we reconstructed residential address histories for 14 027 pregnant women, 10 028 of whom had gaps and overlaps in their address periods corrected. We assigned life stage-specific exposure to ∼92% of women.

Accounting for residential mobility, mean PM_10_ exposure during pregnancy (*n *=* *12 752) across all women was 32.6  μg/m^3^ (S.D. = 3.0  μg/m^3^) and 31.4  μg/m^3^ (S.D. = 2.6  μg/m^3^) during infancy (*n *=* *12 525); 3414 women included in the study (24%) changed address during pregnancy and first year of life of their baby. The majority of those moves occurred after birth: 6.7% (*n *=* *937) of mothers moved during pregnancy and 18.0% (*n *=* *2477) of mothers moved during infancy of their baby. Among those who moved, 95% moved once, 4.5% moved twice and 0.5% moved three times. The average number of addresses women lived at was 1.3.

**Figure 2.**
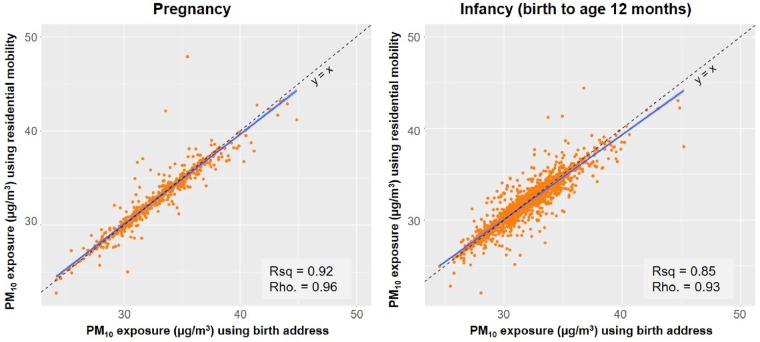
Estimated PM_10_ exposure at residential address comparing birth address only with residential mobility during pregnancy and infancy. Solid line is the linear regression fit line and the dashed line is identity.

In comparing estimated PM_10_ exposures using address at birth and those accounting for residential mobility (Figure 2), differences were up to 26% during pregnancy, ranging from −5.3  μg/m^3^ to 12.4  μg/m^3^ (5th to 95th percentileile, −1.1 to 1.0  μg/m^3^), and up to 27% during infancy, ranging from −7.2  μg/m^3^ to 7.6  μg/m^3^ (5th to 95th percentile, −1.7 to 1.3  μg/m^3^). Using address at birth explained 92% of the variability in estimated PM_10_ exposures for pregnancy and 85% for infancy, compared with using residential mobility. Most individual exposure changed by <5% (95% and 90% for pregnancy and infancy, respectively) despite the relatively large changes that were seen both in pregnancy and infancy (Figure 2).

## Discussion

Our study used a cohort contact database to clean and reconstruct residential address histories for cohort members for a rich and well-characterized birth cohort, ALSPAC. To do so we developed ALGAE, an automated protocol to assess historical exposure to air pollution for members of longitudinal cohort studies. The protocol could easily be adapted to support different environmental pollutants and is intended as a generic and re-usable tool to link environmental exposures to cohort studies by: (i) reconstructing residential address histories; (ii) assigning exposure estimates to cohort participants based on residential address; and (iii) aggregating exposure estimates over different time periods such as pregnancy, infancy and childhood through to adolescence. We used as an example an English birth cohort, but ALGAE is readily transferable and adaptable to other settings with similar demands on reconstructing address histories.

By correcting address histories for 69% of participants, we were able to assign daily exposure estimates to 92% of all women (*n *=* *12 905). We were limited in that we could not include women who moved outside the study area (∼3.5%) as we did not have exposure data outside the modelling domain. The number of women changing address during pregnancy within the study area (∼7%) was consequently lower compared with other studies. In the UK, Hodgson *et al.* (2015)^21^ identified 24% movers in a study of 5399 pregnant women in the North East of England. Canfield *et al.* (2006),^13^ for example, reported that out of 1085 mothers in a Texan case-control study on birth defects, ∼30% of mothers changed address during pregnancy. Chen *et al.* (2010)^14^ found that in a New York birth cohort study, 16.5% of expecting mothers moved. The consensus across these studies was that mobility varied significantly by maternal age and socioeconomic deprivation, with older, more affluent mothers less likely to move. Such sociodemographic data were not analysed as part of the present study.

When comparing estimated exposures obtained using the reconstructed address histories with those obtained using address at birth for the whole duration of pregnancy, exposure estimates varied little overall, with differences between the two methods for the majority of individuals smaller than 5%. This is consistent with previous studies which only reported small changes between exposures at birth compared with those taking into account residential mobility. At the extreme end, however, we observed differences in exposure estimates of up to 26% during pregnancy and up to 27% during the first year of the child’s life. The extent of exposure misclassification introduced by ignoring these observed residential mobility patterns. and instead assigning exposure to the address at delivery. will depend on the degree of spatial and temporal variability in the exposures and the geographical resolution at which they are estimated.^21^ Chen *et al.* (2010),^14^ for example, noted that the majority of moves during pregnancy occurred over short distances (median distance 4 km) and, therefore, exposure estimates did not change substantially when compared with those obtained only at place of birth. They concluded that the level of observed agreement is likely to decrease for studies using higher resolution exposure estimates. We did not have information about the average distance women moved as part of this study, to preserve the un-identifiability of study participants. Our primary interest was in air pollution exposure, but derived distance output could be added to the ALGAE code base if required for other studies, subject to ethical approval.

**Figure 3.**
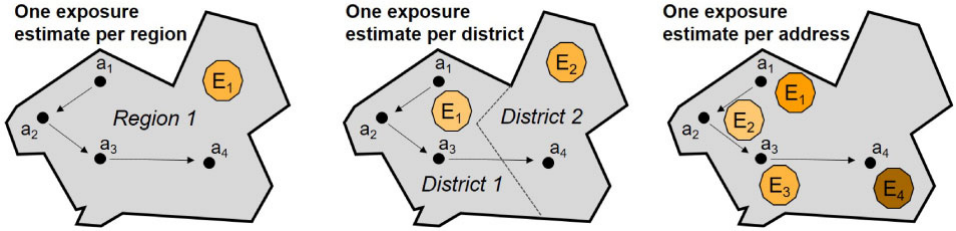
The effect of geographical resolution on exposure assessments that take account of mobility: left—low-resolution exposure (E) will result in same exposure estimates across all addresses (a); middle—medium resolution exposure will have low impact on exposure misclassification; right—exposure misclassification is potentially substantial if high-level exposure (e.g. address-specific) is available.

The granularity of exposure assignment relates to the type of geographical unit used to represent the address, from areas such as regions and districts to addresses (points with X/Y coordinates). Using address locations is especially important in studies where local, discrete air pollution sources (e.g. roads, industrial stacks) are modelled, as exposure estimates may vary substantially over short distances from sources, as shown in Figure 3. In ALSPAC, for example, we modelled PM_10_ emissions from >1500 road sources, so it was important to have a complete record of spatially resolved address locations for exposure assignment.

Another consideration is the temporal resolution of the exposure data. For this study we computed daily exposure estimates for each address. Reconstructing address histories therefore may only be worthwhile if the exposure modelling is sufficiently granular relevant to the exposure window, such as daily or weekly averages for pregnancy exposure.

Our study is the largest to date to explore the effect of residential mobility during pregnancy on exposure misclassification. A considerable strength is the availability of daily exposure estimates at each residential address. By developing ALGAE, we were able to reconstruct residential mobility and assign trimester-specific exposure estimates for 12 752 pregnancies. The use of ambient air pollution exposure estimates at a residential address, however, does not take account of personal activity patterns of individuals or air pollution from indoor sources. The results presented here, therefore, are only a proxy for personal exposure which might vary substantially by individual, depending on daily activity or occupation.

Our study highlights the need for temporally and spatially detailed information on residential location in environmental epidemiological studies. Residential location is often used to assign spatially variable environmental risk factors as a proxy for individual exposure. Our case study demonstrates how the cohort contact database can be used and enhanced to achieve a consecutive temporal address history in cases where recalled address information is not available. We were not able to compare our results with those from recalled address histories as this information was not available in ALSPAC. Previous studies have, however, found an up to 90% agreement between recalled address histories and those obtained from public record databases.^22^ Also, we assume that the start date of an address period is a stronger signal than the end date of the address period, a decision which was taken in collaboration with ALSPAC. The ALGAE code base has the flexibility to allow alterative assumptions which might be more appropriate within other cohort settings.

Our paper highlights ways to improve exposure assessment in cohort studies, where exposures relate to aspects of the environment associated with location of residence. This includes exposures related to the physical environment such as air and noise pollution, as well as social factors such as access to health care services or area-level deprivation. Brokamp *et al.* (2016),^23^ for example, showed that using a single address at one point in time to assign environmental exposures, and other place-based factors such as socioeconomic status, can result in differential exposure misclassification leading to bias towards the null. The ability to identify frequent moves may also be important for other studies; for example looking at mental health outcomes, as frequent moving in childhood has been associated with poorer mental health in adulthood.^24^

In conclusion, whereas ignoring residential changes during pregnancy may on average have a relatively small effect on environmental exposure estimates at residence, at least in the ALSPAC cohort studied here, for some individuals there may be quite marked exposure misclassification which could introduce bias into the study through either random or systematic errors (or both). Differences in exposure are likely to be larger in more mobile populations. Our bespoke software to assign air pollution data to residential histories, dealing with gaps, overlaps and errors in address records, offers a ready solution to link environmental data to individuals in longitudinal cohort studies. Its generic code base makes the ALGAE re-usable for other cohort studies in the UK and internationally, providing an accessible and low-cost means to enhance such studies with environmental exposure data.

## Funding

This work was supported by the UK Medical Research Council and the Wellcome Trust (grant ref.: 102215/2/13/2) and the University of Bristol, who provide core support for ALSPAC. The work presented here was specifically funded by the UK Medical Research Council: ‘Effects of early life exposure to particulates on respiratory health through childhood and adolescence: ALSPAC Birth Cohort Study’ (grant ref: G0700920). We thank Bristol City Council for providing data on traffic flows/speeds and emission rates for the ALSPAC study area. P.E. acknowledges support of the National Institute for Health Research (NIHR) Imperial Biomedical Research Centre and the NIHR Health Protection Research Unit in Health Impact of Environmental Hazards (HPRU-2012–10141). The work of the UK Small Area Health Statistics Unit is funded by Public Health England as part of the MRC-PHE Centre for Environment and Health, funded also by the UK Medical Research Council (MR/L01341X/1).

## References

[1] Gehring U, Gruzieva O, Agius RM et al Air pollution exposure and lung function in children: the ESCAPE Project. Environ Health Perspect 2013;121:1357–64. 24076757 10.1289/ehp.1306770 PMC3855518

[2] Morales E, Garcia-Esteban R, de la Cruz OA et al Intrauterine and early postnatal exposure to outdoor air pollution and lung function at preschool age. Thorax 2015;70:64–73. 25331281 10.1136/thoraxjnl-2014-205413

[3] Tischer C, Gascon M, Fernandez-Somoano A et al Urban green and grey space in relation to respiratory health in children. Eur Respir J 2017;49:1502112. 28642307 10.1183/13993003.02112-2015

[4] Dadvand P, Parker J, Bell ML et al Maternal exposure to particulate air pollution and term birth weight: a multi-country evaluation of effect and heterogeneity. Environ Health Perspect 2013;121:367–73. 10.1289/ehp.1205575 PMC3621183 23384584

[5] Porta D, Narduzzi S, Badaloni C et al Air pollution and cognitive development at age 7 in a prospective Italian birth cohort. Epidemiology 2016;27:228–36. 26426942 10.1097/EDE.0000000000000405

[6] Bowatte G, Lodge C, Lowe AJ et al The influence of childhood traffic-related air pollution exposure on asthma, allergy and sensitization: a systematic review and a meta-analysis of birth cohort studies. Allergy 2015;70:245–56. 25495759 10.1111/all.12561

[7] MacIntyre EA, Gehring U, Molter A et al Air pollution and respiratory infections during early childhood: an analysis of 10 European birth cohorts within the ESCAPE project. Environ Health Perspect 2014;122:107–13. 24149084 10.1289/ehp.1306755 PMC3888562

[8] Guxens M, Garcia-Esteban R, Giorgis-Allemand L et al Air pollution during pregnancy and childhood cognitive and psychomotor development: six European birth cohorts. Epidemiology 2014;25:636–47. 25036432 10.1097/EDE.0000000000000133

[9] Pedersen M, Giorgis-Allemand L, Bernard C et al Ambient air pollution and low birthweight: a European cohort study (ESCAPE). Lancet Respir Med 2013;1:695–704. 24429273 10.1016/S2213-2600(13)70192-9

[10] Ritz B, Wilhelm M, Hoggatt AJ, Ghosh JK. Ambient air pollution and preterm birth in the environment and pregnancy outcomes study at the University of California, Los Angeles. Am J Epidemiol 2007;166:1045–52. 17675655 10.1093/aje/kwm181

[11] Smith RB, Fecht D, Gulliver J et al Impact of London's road traffic air and noise pollution on birth weight: retrospective population based cohort study. BMJ 2017;5:j5299. 10.1136/bmj.j5299 PMC5712860 29208602

[12] Bell ML, Belanger K. Review of research on residential mobility during pregnancy: consequences for assessment of prenatal environmental exposures. J Expo Sci Environ Epidemiol 2012;22:429–38. 22617723 10.1038/jes.2012.42 PMC3543155

[13] Canfield MA, Ramadhani TA, Langlois PH, Waller DK. Residential mobility patterns and exposure misclassification in epidemiologic studies of birth defects. J Expo Sci Environ Epidemiol 2006;16:538–43. 16736057 10.1038/sj.jes.7500501

[14] Chen L, Bell EM, Caton AR, Druschel CM, Lin S. Residential mobility during pregnancy and the potential for ambient air pollution exposure misclassification. Environ Res 2010;110:162–68. 19963212 10.1016/j.envres.2009.11.001

[15] Madsen C, Gehring U, Walker SE et al Ambient air pollution exposure, residential mobility and term birth weight in Oslo, Norway. Environ Res 2010;110:363–71. 20227069 10.1016/j.envres.2010.02.005

[16] Fell DB, Dodds L, King WD. Residential mobility during pregnancy. Paediatr Perinat Epidemiol 2004;18:408–14. 15535816 10.1111/j.1365-3016.2004.00580.x

[17] Lupo PJ, Symanski E, Chan W et al Differences in exposure assignment between conception and delivery: the impact of maternal mobility. Paediatr Perinat Epidemiol 2010;24:200–08. 20415777 10.1111/j.1365-3016.2010.01096.x

[18] Gulliver J, Elliot P, Henderson J et al Local- and regional-scale air pollution modelling (PM_10_) and exposure assessment for pregnancy trimesters, infancy, and childhood to age 15 years: Avon Longitudinal Study of Parents And Children (ALSPAC). Environ Int 2018;113:10–19. 29421397 10.1016/j.envint.2018.01.017 PMC5907299

[19] Boyd A, Golding J, Macleod J et al Cohort Profile: The ‘Children of the 90s’—the index offspring of the Avon Longitudinal Study of Parents and Children. Int J Epidemiol 2013;42:111–27. 22507743 10.1093/ije/dys064 PMC3600618

[20] Garwood K. The ALGAE Protocol - Algorithm for Generating Address Histories and Exposures. London: Small Area Health Statistics Unit, 2016.]

[21] Hodgson S, Lurz PW, Shirley MD, Bythell M, Rankin J. Exposure misclassification due to residential mobility during pregnancy. Int J Hyg Environ Health 2015;218:414–21. 25845985 10.1016/j.ijheh.2015.03.007

[22] Wheeler DC, Wang A. Assessment of residential history generation using a public-record database. Int J Environ Res Public Health 2015;12:11670–82. 26393626 10.3390/ijerph120911670 PMC4586699

[23] Brokamp C, LeMasters GK, Ryan PH. Residential mobility impacts exposure assessment and community socioeconomic characteristics in longitudinal epidemiology studies. J Expo Sci Environ Epidemiol 2016;26:428–34. 26956935 10.1038/jes.2016.10 PMC4913165

[24] Webb RT, Pedersen CB, Mok PL. Adverse outcomes to early middle age linked with childhood residential mobility. Am J Prev Med 2016;51:291–300. 27288289 10.1016/j.amepre.2016.04.011 PMC4982753

